# A Treatise on the Surgery of the Alimentary Canal

**Published:** 1897-06

**Authors:** 


**A Treatise on the Surgery of the Alimentary Canal.** By A. ERNEST MAYLARD, M.B., B.S. Pp. xxiv., 724. London: J. & A. Churchill. 1896.

It is not easy to write a review of this work: it is at once so praiseworthy and so disappointing. It is praiseworthy in this, that it proves great industry in reading; it is disappointing because it is incomplete in the presentation of facts and without discrimination in practical advice. Not enough has been read to provide a full summary, and not enough practice has been had to provide definite and discriminative advice. Thus the work is of little use to the expert and confusing to the tiro.

The arrangement of matter is not altogether satisfactory. Chapters are arranged apparently according to their length, and their length alone. Thus, chapter VII. concerns "Tumours: malignant—carcinoma and sarcoma;" chapter VIII. "Carcinoma (*continued*)." Chapter IX. deals with "Non-malignant or cicatricial stricture;" chapter X. with "Cicatricial stricture (*continued*)." And so it is all through. Appendicitis has one chapter to itself and three "*continueds*." A book on intestinal surgery is not a serial novel, where the length of the chapter is regulated by the size of the magazine: on such a subject as this scientific arrangement and sequence should alone be permitted to rule.

As an example of the method of the work we might fairly select Malignant Disease of the Œsophagus, in Chapters vii. and viii., to the titles of which we have called attention. As to treatment, we find firstly some advice as to foods, secondly the use of tubage, and thirdly "for separate and fuller consideration the question of operation" is left. Here however we have mention made of gastrostomy, and all that we are told of its value (p. 81) is that "David Newman records four consecutive successful cases." Then follows this sentence: "Contrasting, however, the operation successfully performed with the alternative of permanent tubage, it must be confessed that in the majority of instances the patient with a tube in the œsophagus is, in various ways, in a more comfortable position than one with an artificial orifice in the stomach." This may be true, but the author provides no facts in support of his argument. He makes no reference to vital facts in selection of methods. Œsophagostomy and gastrostomy are classed as "the two operations worthy most consideration." "The former operation," we are told, "is performed when the disease is located high up, and the opening in the œsophagus being thus below the seat of obstruction, a feeding tube can be easily passed from the wound into the stomach. Occasionally the œsophagus is opened above the level of the disease, the object then being to facilitate the passage of a tube which otherwise would be conducted with pain or difficulty through the natural orifices." Statements such as these, directly contravening the enlightened and experienced surgery of the day, should be supported by something more than facile remarks of the author. As compared with the four consecutive successful cases of gastrostomy of David Newman, at least something should have been quoted in favour of œsophagostomy above the stricture.

Some of the chapters in the book are decidedly below the level we should expect in a modern work. The worst are those dealing with intestinal obstruction. In the treatment not a word is said about intestinal drainage, which most surgeons regard as of some importance. More than two of the four pages given to a discussion of the treatment are occupied with a table, and explanation of some thirty cases, culled chiefly from the *Lancet* and the *British Medical Journal*, of which the author makes the *naif* remark: "It is possible that every successful case is published, but it is certain that every unsuccessful one is not." For statistics of operations for intestinal obstruction, Mr. Maylard should go to Hospital Reports and not to Medical Journals. Not every hospital surgeon now-a-days publishes every successful case of intestinal obstruction which he operates upon. Similar criticism might be made of the author's handling of other varieties of obstruction. Everywhere he has missed some crucial point in treatment: nowhere can he be relied on as a safe guide. Most markedly is this seen in intussusception and in volvulus.

Our careful study of this work leads to the conclusion that it is worth reading, but not safe to follow. It is worth reading because it brings together many stray facts and observations, gathered in wide if not exhaustive study: it is not safe to follow because it does not reflect the best work of the best surgeons, and provides no original or well-supported advice from the author himself.

